# Retracted: Analysis of Pull-In Instability of Geometrically Nonlinear Microbeam Using Radial Basis Artificial Neural Network Based on Couple Stress Theory

**DOI:** 10.1155/2018/4030684

**Published:** 2018-05-02

**Authors:** Computational Intelligence and Neuroscience

Computational Intelligence and Neuroscience has retracted the article titled “Analysis of Pull-In Instability of Geometrically Nonlinear Microbeam Using Radial Basis Artificial Neural Network Based on Couple Stress Theory” [1]. The article was found to contain a substantial amount of material from the following published article: Mohammad Heidari, Yaghoub Tadi Beni, and Hadi Homaei, “Estimation of Static Pull-In Instability Voltage of Geometrically Nonlinear Euler-Bernoulli Microbeam Based on Modified Couple Stress Theory by Artificial Neural Network Model,” Advances in Artificial Neural Systems, vol. 2013, Article ID 741896, 10 pages, 2013. doi:10.1155/2013/741896 [2]. In addition, Figures 17 and 18 in [1] are similar to Figures 9 and 11 in [2]. Dr. Mohammad Heidari did not agree with this retraction, while Drs. Ali Heidari and Hadi Homaei were not reachable.

## References

[1] Heidari M., Heidari A., Homaei H. (2014). Analysis of pull-in instability of geometrically nonlinear microbeam using radial basis artificial neural network based on couple stress theory.

[2] Heidari M., Tadi Beni Y., Homaei H. (2013). Estimation of Static Pull-In Instability Voltage of Geometrically Nonlinear Euler-Bernoulli Microbeam Based on Modified Couple Stress Theory by Artificial Neural Network Model.

